# Photoelectrochemical detection of ultra-trace fluorine ion using TiO_2_ nanorod arrays as a probe

**DOI:** 10.1039/c9ra04367e

**Published:** 2019-08-27

**Authors:** Yongzhao Su, Duotian Chen, Siyuan Yang, Shengsen Zhang, Yingju Liu, Yueping Fang, Qiao Zhang, Feng Peng

**Affiliations:** School of Chemistry and Chemical Engineering, South China University of Technology Guangzhou 510640 China; College of Materials and Energy, South China Agricultural University Guangzhou 510643 China Zhangss@scau.edu.cn; School of Chemistry and Chemical Engineering, Guangzhou University Guangzhou 510006 China fpeng@gzhu.edu.cn

## Abstract

A photoelectrochemical (PEC) method based on the etching reaction of F ions on the surface of TiO_2_ nanorod arrays (TNRs) was proposed for the high sensitivity and selectivity detection of F ions. With the increase of F ion concentration, the surface etching reaction on TNR becomes more intense, resulting in the increased number of surface active sites, the reduction of electron transfer resistance, and the increase of photocurrent density. The prepared TNRs as a PEC probe exhibits a good linear relationship between photocurrent increment and the logarithm of F ion concentration in the range from 0.05 to 1000 nM with an ultra-trace detection limit of 0.03 nM for F ion detection.

## Abstract 2

A photoelectrochemical (PEC) method based on the etching reaction on TiO_2_ nanorod arrays is proposed for detection of F ions.

## Graphical abstract

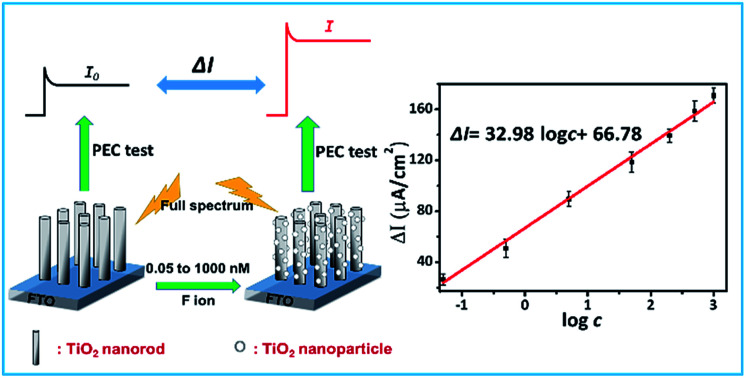

## Introduction

The fluorine ion (F ion), as the smallest anion among the anions,^1^ has gained significant attention due to its important role in health and environmental issues, such as dental health,^2^ orthodontic appliances,^3^ osteoporosis^4^ and pharmaceutical agents.^5^ In the normal concentration range, fluorine could be easily absorbed by the human body, but excretion is slow.^6^ The permissible limit of F ion in drinking water recommended by the World Health Organization (WHO) is 1.5 ppm.^7^ However, when it exceeds this limit, fluorosis^8^ may occur, which is harmful to human health, such as speckle tooth and fluorosis of bone. Therefore, there is an urgent need to explore a method for effectively detecting F ions. Many technologies have been explored for the detection of F ions, such as ion selective electrodes, high-performance liquid chromatography, atomic absorption spectrometry, and resonance Rayleigh scattering. However, some sensing systems are inappropriate for wide application due to instability of detection,^9^ expensive instruments,^10^ complex sample handling,^11^ or specific detection conditions.^12^ It is of great interest and importance to look for a simple and effective material and method for highly sensitive and selective detection of F ions.

It is well known that the photoelectrochemical (PEC) detection method is based on the photo-to-charge conversion process.^13^ The light and electricity are used for the sensor excitation and detection respectively,^14^ indicating that PEC detection technology has high sensitivity.^15^ Moreover, the PEC method has attracted widespread concern because of its low cost and convenient.^16–18^ Recently PEC detection based on TiO_2_ nanorod arrays (TNRs) has attracted the attention of many researchers because TNRs-based materials have unique chemical, physical, photocatalytic properties^19,20^ and the advantages of photogenerated charge separation and transport.^21^ For example, Feng *et al.*^22^ exploited TiO_2_/CdS nanorod array as a PEC sensor to detect Cu^2+^ in human serum samples. Wang *et al.*^23^ prepared the hydrogenated TiO_2_ nanorod film to detect chemical oxygen demand (COD) based on its visible light photoelectrochemical properties. Samir Kumar *et al.*^24^ synthesized Ag–TiO_2_ nanorod SERS substrates to sensitively detect dye molecules under ultra-violet (UV) light irradiation. However, the determination method of F ion based on PEC technology has never been reported so far.

**Scheme 1:**
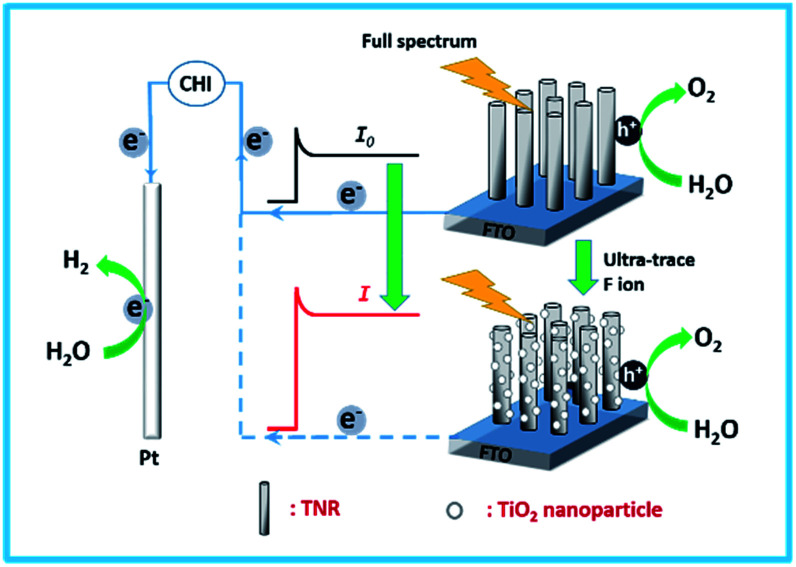
Schematic illustration of the photoelectrochemical probe for the detection of F ion.

Herein, TNRs were prepared by a hydrothermal method as a PEC sensor for F ion determination. In the presence of F ion, the surface of the TNRs is etched, which results in higher photocurrent density due to more surface-active sites and smaller electron transfer resistance (Scheme 1). Based on this, a simple, economical and efficient PEC detector for detection F ion was designed. Under optimal test conditions, an ultra-sensitive PEC sensor for F ion was established with an ultra-low detection limit of 0.03 nM.

## Experimental section

### Preparation of TiO_2_ nanorod arrays (TNRs)

30 mL of deionized water was mixed with 30 mL of concentrated hydrochloric acid (36.5 wt%) for 5 min and transferred to a 100 mL Teflon-lined stainless steel autoclave, followed by the slow addition of 0.8 mL of titanium butoxide (98%, Aladdin) and stirring for another 5 minutes. Then, a piece of cleaned FTO substrate (6 × 3 cm^2^, 7 Ω cm^−2^, Hartford Glass Company) was placed into the mixture solution at an angle against the wall with the conducting side facing down. The hydrothermal synthesis was conducted at 170 °C for 3, 5, 7, and 9 hours, respectively. The resultant samples were taken out, rinsed thoroughly with deionized water, and allowed to dry in air. The as-prepared samples were denoted as TNRs-*t*, where *t* indicated the hydrothermal time (hours).

### Characterizations

The morphology of the samples were investigated using a scanning electron microscopy (SEM, FEI Quanta 200 FEG). An X-ray diffractometer (D/max-IIIA) with Cu Kα radiation was used to analyze the X-ray diffraction (XRD) over the 2*θ* range of 10 to 80°. X-ray photoelectron spectroscopy (XPS) was collected on Escalab 250 Xi spectrometer with Al Kα radiation. UV-visible absorption spectra were obtained using a JASCO V-560 UV-vis spectrophotometer.

### Photoelectrochemical detection of F ion

The photocurrent measurements were recorded in the electrolytic solution of 0.1 M Na_2_SO_4_ using the TNRs, Ag/AgCl and Pt mesh as the working, reference and counter electrodes, respectively. The full spectrum irradiation was provided by the Xe lamp with 150 mW cm^−2^. For PEC detection of F ion, the photocurrents of TNRs were measured before and after immersion in the NaAc–HAc buffer solution with different concentrations of NaF solution.

## Results and discussions

### Structure characterizations and photoelectrochemical behaviors of TNRs

**Fig. 1:**
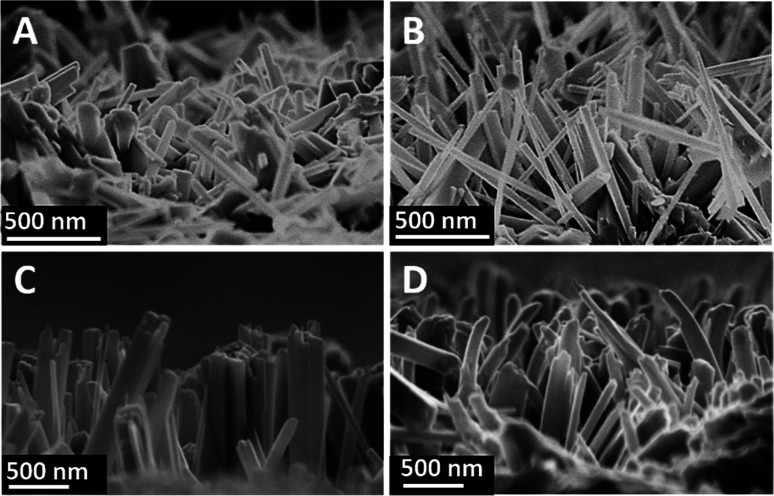
SEM images of the TNRs prepared by different hydrothermal time: (A) 3 hours, (B) 5 hours, (C) 7 hours, and (D) 9 hours.

Fig. 1 shows the SEM images of a cross-sectional view of the TNRs prepared by different hydrothermal time. It can be seen that the TNRs were almost vertically loaded onto the FTO substrate. At the beginning of the growth (Fig. 1A), the nanorod array is relatively short and thin. Fig. 1B and C show the TNRs with the average length of about 700 nm, but the nanorod diameter of TNRs-7 is larger than that of TNRs-5. However, the TNRs became irregular when the hydrothermal time was extended to 9 hours, as shown in Fig. 1D. In addition, the nanorods of TNRs-7 and TNRs-9 were assembled together significantly, suggesting a smaller specific surface area of exposure. Therefore, TNRs-5 has a relatively high surface area, suggesting it has a better PEC response.

**Fig. 2:**
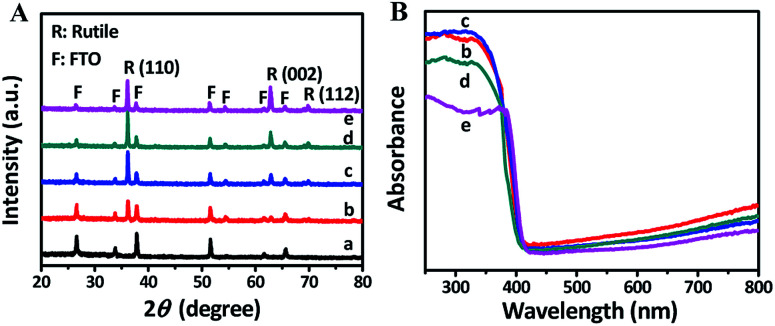
XRD (A) and UV-vis absorption (B) patterns of samples: (a) FTO, (b) TNRs-3, (c) TNRs-5, (d) TNRs-7 and (e) TNRs-9.

The XRD patterns of FTO and TNRs are shown in Fig. 2A. Compared with the FTO, three sharp peaks at 35.52°, 62.26°, 69.28° are observed in the TNRs, assigning to the (110), (002) and (112) planes of rutile TiO_2_ (JCPDS no. 88-1175), respectively. Therefore, the rutile TiO_2_ with high crystallinity was successfully synthesized by the hydrothermal method. The UV-vis absorption spectra of TNRs prepared at the different hydrothermal time were analyzed. Fig. 2B shows the absorption intensity of TNRs in the ultraviolet region decreases gradually with the hydrothermal time increasing. Compared with the other hydrothermal time, the TNRs-5 achieves the maximum absorption intensity of ultraviolet light, which might be due to the larger effective surface area. This result is consistent with the results from SEM images.

**Fig. 3:**
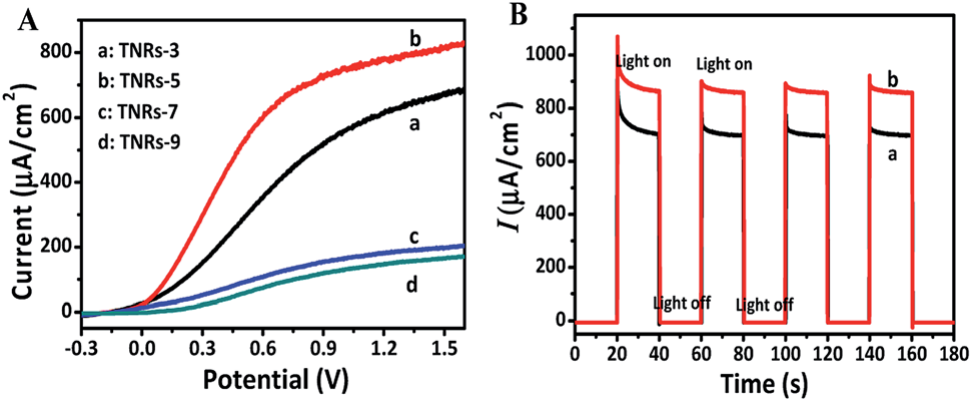
Linear sweep voltammograms (A) of TNRs prepared by different hydrothermal time. Photocurrent intensities (B) of TNR-5 before (a) and after (b) immersion in a solution of 500 nM F ion.

The linear sweep voltammograms (LSV) of TNRs were carried out in 0.1 M Na_2_SO_4_ under the irradiation of UV-vis light (150 mW cm^−2^) (Fig. 3A) at the potential from −0.3 V to 1.6 V (*vs.* Ag/AgCl). The result shows that the photocurrent increases monotonically with the increase of applied potential, and reaches a saturation photocurrent at about 0.8 V. Generally, the PEC sensor generally chooses the saturated photovoltage as the detection bias, which can reduce the influence of bias voltage.^19,20^ Therefore, the potential for 0.8 V was selected as the bias voltage for the measurement of photocurrent intensities. The TNRs-5 possesses better photoelectrochemical properties than the other TNRs, which agrees with the result of the UV-vis absorption spectrum. Fig. 3B displays the photocurrents diagram of TNR-5 before and after immersion into a solution of 500 nM F ion with four “light-on” and “light-off” cycles. There is no measurable current was observed in the dark for the TNRs-5 sample. Under light irradiation, the photocurrent momentarily exceeds 800 µA cm^−2^ and then reaches a steady state after the second “Light on/off” cycle. After TNRs immersed in the F ion solution of 500 nM for 4 min, the detected photocurrent intensity increases obviously, which represents the TNRs-5 could be used as the PEC sensor for detecting F ion.

### Optimization of F ion detection conditions

**Fig. 4:**
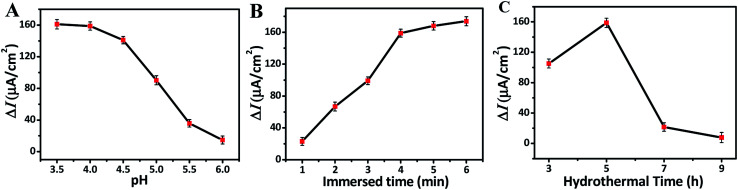
Effect of the pH (A), immersed time (B), and hydrothermal time (C) on the photocurrent change.

In order to obtain the optimal photoelectrochemical detection performance, the pH value of NaAc–HAc buffer solution and the effects of immersed, hydrothermal time on F ion detection were analyzed. The sensitivity as an important density of TNRs before and after immersing in NaF solution, respectively. Fig. 4A shows that the Δ*I* does not change obviously in the range of pH 3.5–4.0, but the photocurrent decreases rapidly with the increase of pH from 4.0 to 6.0, indicating that the PEC sensor has good sensitivity for F ion detection at pH 3.5–4.0. Fig. 4B shows the Δ*I* of TNRs increased with the increasing immersion time. However, the Δ*I* increases slightly when the immersion time exceeds 4 min. In addition, the choice of 4 min can reduce the time cost. Therefore, pH of 4.0 and immersion time of 4 min were selected as the optimal detection conditions to study the effect of hydrothermal time on the detection of F ion. Under the optimal detection conditions, the samples prepared with different hydrothermal time were used to detect the F ion solution of 500 nM. Fig. 4C displays the value of Δ*I* reaches the maximum when the hydrothermal time is 5 hours. That is to say, TNRs-5 has the largest sensitivity for F ion detection, which is consistent with the results of structural characterization.

**Fig. 5:**
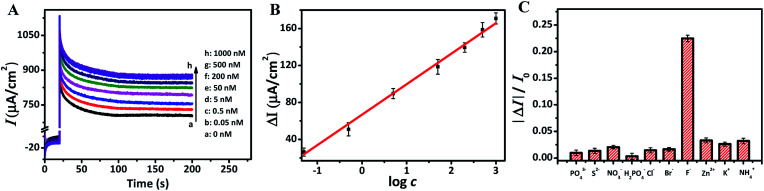
Photocurrent intensities (A) of TNRs-5 under 0.8 V bias voltage (*vs.* Ag/AgCl) after immersion into F ion solution of different concentrations. The calibration line of relative photocurrent increase with the logarithm of the concentration of F ion (B). Relative photocurrent change of the TNRs-5 in 0.1 M Na_2_SO_4_ solution containing 5 µM interfering ions (C).

To explore the relationship between F ion concentrations and photocurrent densities, the photocurrent intensities of TNRs-5 in different concentrations of NaF solution were measured. The photocurrent density of TNRs-5 increases from 733 to 878 µA cm^−2^ as the concentration of F ion increases, which indicates a quantitative relationship between the concentration of F ion and the photocurrent density of TNRs (Fig. 5A). Furtherly, the calibration line of Δ*I* with the logarithm of the concentration of F ion (log *c*) was carried out. As shown in Fig. 5B, there is a good linear relationship (*R*^2^ = 0.995) between the Δ*I* and the log *c* (Δ*I* = 32.98 log *c* + 66.78) in the range from 0.05 to 1000 nM of F ion concentrations. The detection limit is evaluated to be 0.03 nM using the 3*σ*/*S* method. The result reveals that the TNRs can be used for measuring F ion accurately. Also, to verify the effectiveness of this method, the selectivity of the TNRs as a PEC sensor was proven by introducing 5.0 µM different anions or cations such as PO_4_^3−^, S^2−^, NO_3_^−^, H_2_PO_4_^−^, Cl^−^, Br^−^, Zn^2+^, K^+^ and NH_4_^+^ into 0.5 µM F ion buffer solution. As shown in Fig. 5C, the relative photocurrent (Δ*I*/*I*) of TNRs-5 in F ion solution is significantly higher than that of 10-fold concentration of other interfering ions. The result indicates that TNRs used as a PEC sensor exhibits high selectivity for detection of ultra-trace F ion. In addition, the experimental data about the repeatability of the TNRs can be seen in Fig. 5B and C, in which the error bars were obtained by testing 3 times with parallel samples. Fig. 5A shows good stability of the TNRs probe with stable photocurrent after 100 s, which is long enough to determine the concentration of fluoride ion, comparing with the detecting of glutathione with the steady signal within only 15 s.^25^Table 1 shows the recently reported results for the detection of F ion. Compared with other methods, the TNRs-5 photoelectrode sensor has superior sensitivity and selectivity, which can realize the detection of trace F ion, and has an obvious advantage.

**Table 1:** An overview of recently reported methods for the detection of F ion

Method applied	Materials used	LDR^a^ (µM)	LOD^b^ (µM)	Ref.
Colorimetry and fluorescence	1*H*-imidazo[4,5-*b*]phenazine derivative	0–110	6.2	26
Colorimetry and fluorescence	BODIPY-containing conjugated polymer	0–500	0.523	1
Colorimetric	Perylene-3,4:9,10-tetracarboxylic bisimide	0–10.00	Unknown	27
Plasma processes	ICPMS/MS	5.27–527.00	0.2	9
Chemodosimeter	Diketopyrrolopyrrole derivative	0–3.00	0.2	28
Resonance Rayleigh scattering	Graphene oxide/nanogold	0.06–13	0.03	29
This method	TNRs	5 × 10^−5^ to 1.00	3 × 10^−5^	This work

a Linear dynamic range (LDR).

b Limit of detection (LOD).

For the practical application demonstration of the probe, the photocurrent responses of the TNRs to ultra-trace F ion in tap water and lake water were tested. After diluted 1000-fold with NaAc–HAc buffer solution, the environmental water samples were spiked with 0.05, 5, 500 nM F ion and assayed using the method. The samples of lake water were centrifuged for 20 min at 10 000 rpm with the centrifugal force of 9920 × *g* to remove any particulate suspension before testing. As shown in Table 2, the photocurrent responses to F ion in tap water and lake water are highly consistent since the recoveries are between 99.1 to 105.2.

**Table 2:** Determination of F ion in real water samples

Sample	Added (nM)	Average Δ*I*/(µA cm^−2^)	Found (nM)	RSD (*n* = 3) (%)	Recovery (%)
Tap water 1	0.05	24.1	0.051	3.5	101.6
Tap water 2	5	89.7	4.954	2.2	99.1
Tap water 3	500	156.1	510.858	2.7	102.2
Lake water 1	0.05	24.6	0.053	4.3	105.2
Lake water 2	5	90.5	5.238	2.7	104.7
Lake water 3	500	156.5	525.291	3.6	105.1

### The detection mechanism on F ion detection

**Fig. 6:**
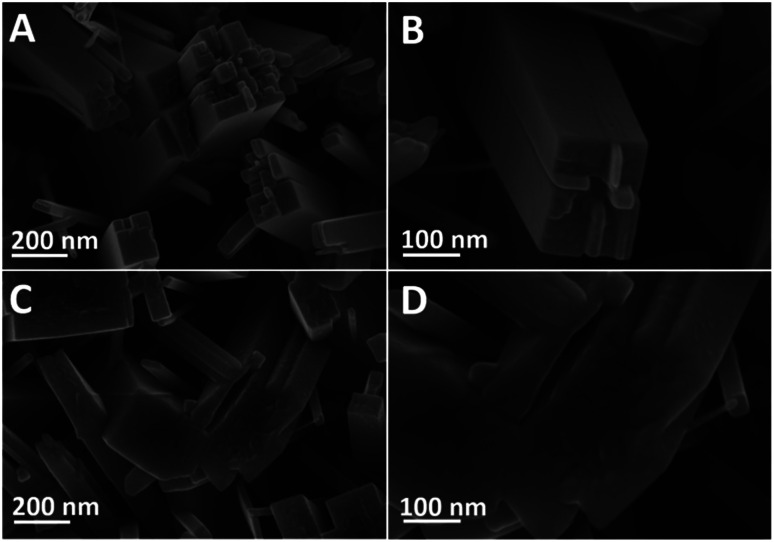
SEM images of the TNRs-5 before (A and B) and after (C and D) immersion in a solution of 1000 nM F ion.

In order to explore the PEC sensing mechanism for F ion, TNRs before and after detection of 1000 nM F ion were characterized. SEM images display the average diameter of the TNRs is about 150 nm, and there is no change before (Fig. 6A and B) and after (Fig. 6C and D) immersed in a solution of 1000 nM F ion. However, the smooth surface (Fig. 6B) of TNRs becomes “countless bumps” (Fig. 6D) after F ion detection due to the corrosion of TiO_2_ by F ion. The formation of the numerous bumps involves three steps.^30^

In the solution of pH = 4.0, F ion is a corrosive chemical. The TiO_2_ can be etched by F ion:

1 TiO_2_ + 6H^+^ + 6F^−^ → H_2_TiF_6_ + 2H_2_O

Subsequently, some H_2_TiF_6_ combines with H_2_O, forming Ti(OH)_4_:

2 H_2_TiF_6_ + 4H_2_O → Ti(OH)_4_ + 6HF

The formed Ti(OH)_4_ turns to TiO_2_ initially, nucleates, and grows into TiO_2_ nanoparticles, *i.e.*, numerous bumps.

3 Ti(OH)_4_ → TiO_2_ + 2H_2_O

The countless bumps significantly increase the effective active sites of the TNRs. It indicates that F ion reacts with TNRs on its surface, resulting in an increase in photocurrent density.

**Fig. 7:**
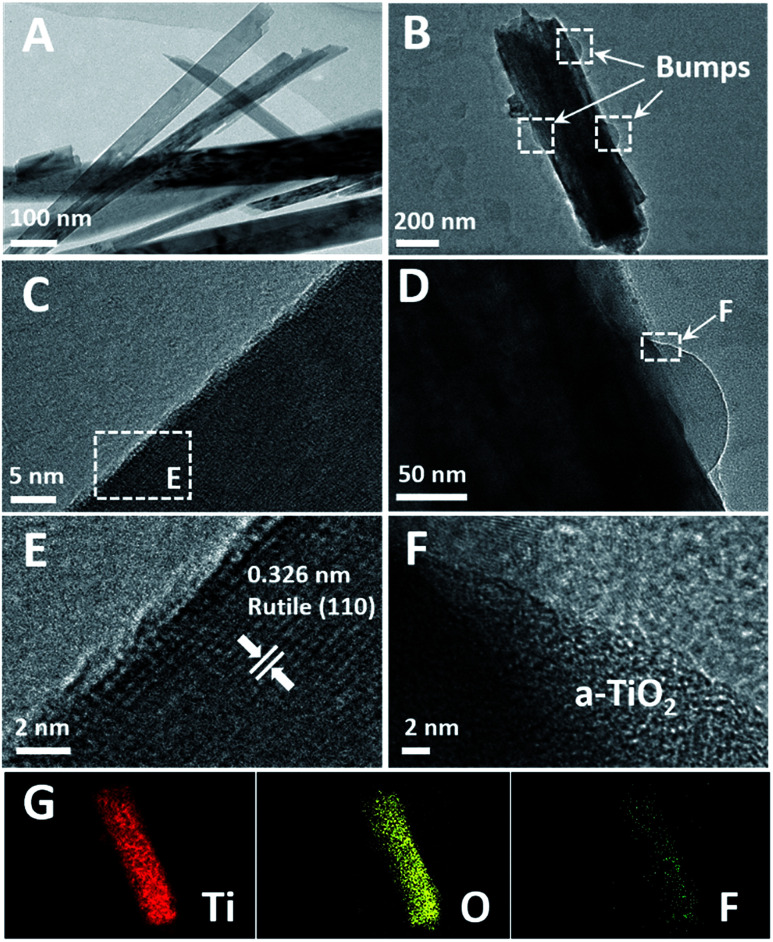
HRTEM images of the TNRs-5 before (A, C and E) and after (B, D and F) immersion in a solution of 1000 nM F ion, elemental mapping (G) of B.

As shown in Fig. 7, TEM images of the TNRs-5 before and after immersion in a solution of 1000 nM F ion are provided. After immersed in the F ion, TNRs-5 was etched to form some bumps on the rod (Fig. 7B and D) compared to the untreated sample (Fig. 7A and C), corresponding to the consequences of SEM images. Fig. 7E displays that the TNRs exhibit a rutile structure, which corresponds to the result from the XRD patterns. However, the bumps on the surface on TNR are amorphous TiO_2_ (a-TiO_2_), which increases the active sites and also be the cause of the increase of response photocurrents.^19,31,32^ In addition, elemental mapping of the TNRs-5 after immersed in a solution of 1000 nM F ion was also characterized. As shown in Fig. 7G and F element on the TNRs-5 is obviously less than Ti and O, which may be residual after cleaning with deionized water, indicating that residual fluorine is not the main reason for the increase of photocurrent.

**Fig. 8:**
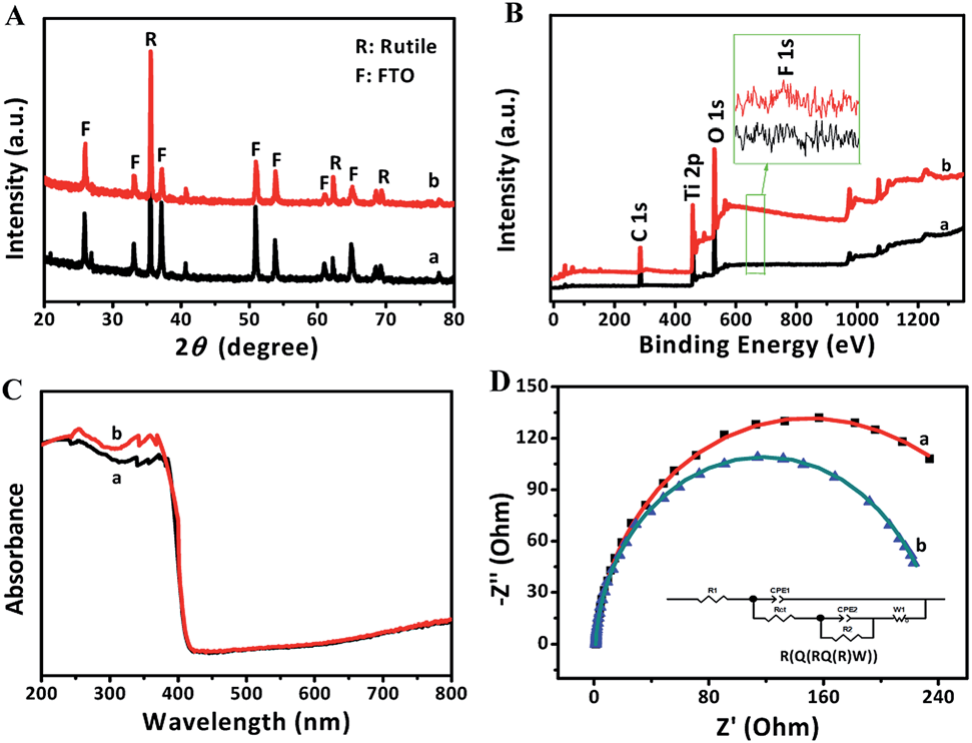
XRD patterns (A), XPS spectra (B), UV-vis absorption spectra (C), EIS Nyquist plots of full under 0.8 V bias voltage and light irradiation (D) of TNRs-5 before (a) and after (b) immersion in a solution of 1000 nM F ion.

Fig. 8A displays that there was no significant change in the XRD characteristic peaks of rutile TiO_2_ in TNRs-5 before and after detection, which means the newly formed numerous bumps on the surface is also rutile TiO_2_ structure. Fig. 8B shows that the peaks of Ti, O, C elements in XPS spectra were detected in both samples. Among them, the C element is derived from the adventitious carbon of the XPS instrument itself.^33^ The peak 688.6 eV, assigned to the F substitution in TiO_2_ lattice,^34^ could not be observed in the spectrum. However, comparing the high-resolution XPS pattern of F 1s before and after immersing in F ion solution, a small peak of F on the surface of TiO_2_ at 684.3 eV is observed.^35^ These results indicate that a small part of F may dope into TNRs-5, which increases the photocurrent by promoting the separation of photogenerated electrons and holes.

After immersed into the F ion solution, TNRs-5 shows a higher absorption ability in the ultraviolet region, and keeps the same absorption ability in the visible light region, as shown in the UV-vis absorption spectra of Fig. 8C. This result suggests that the photocurrent density of TNRs-5 could increase after being immersed in F ion. However, the absorption in the visible-light region has no significant change, revealing the F was not doped into the TiO_2_.^36^ From the EIS Nyquist plots, it can be seen that the arc radius of the used TNRs-5 after detecting F ion is obviously smaller than that of the original TNRs-5, and the calculated value of *R*_ct_ for the both samples is 248.8 Ω cm^−2^ and 223.8 Ω cm^−2^, respectively (Fig. 8D), indicating the enhance of charge transfer efficiency. F ion significantly changes the surface properties of TNRs-5 *via* the improvement of charge transfer resistance and separation efficiency, which is a critical factor for the PEC performance. In addition, the residue of F ion on the surface of TNRs-5 may promote the conductivity of its surface.

## Conclusions

In this work, an efficient and selective PEC sensor was designed to detect F ion based on TNRs photoelectrode. F ion significantly changes the surface properties of TiO_2_*via* the improvement of charge transfer resistance and separation efficiency, which is a critical factor for the PEC performance. The optimal detection conditions, which are the NaAc–HAc buffer solution with pH of 4.0 and the immersion time of 4 min, were recommended. The kind of PEC probe exhibits a good linear relationship between photocurrent increment and the logarithm of F ion concentration in the range from 0.05 to 1000 nM with an ultra-trace detection limit of 0.03 nM for F ion detection, indicating a promising application to the detection of water quality.

## Conflicts of interest

There are no conflicts to declare.

## Supplementary Material
